# Primary Small Cell Neuroendocrine Carcinoma of the Sublingual Gland Treated With Definitive Chemoradiotherapy: A Case Report

**DOI:** 10.1002/ccr3.72565

**Published:** 2026-04-24

**Authors:** Taka‐aki Tokura, Hironari Dehari, Koyo Nishiyama, Sho Miyamoto, Takanori Sasaki, Kazuhiro Ogi, Taro Sugawara, Masanori Someya, Kohichi Takada, Hiroyuki Kitajo, Akihiro Miyazaki

**Affiliations:** ^1^ Department of Oral Surgery Sapporo Medical University School of Medicine Sapporo Japan; ^2^ Department of Surgical Pathology Sapporo Medical University School of Medicine Sapporo Japan; ^3^ Department of Radiology Sapporo Medical University School of Medicine Sapporo Japan; ^4^ Division of Medical Oncology, Department of Internal Medicine Sapporo Medical University School of Medicine Sapporo Japan; ^5^ Sapporo Teine Dental and Oral Surgery Clinic Sapporo Japan

**Keywords:** definitive chemoradiotherapy, extrapulmonary small cell carcinoma, head and neck small cell carcinoma, small cell neuroendocrine carcinoma, sublingual gland

## Abstract

Primary sublingual gland small cell neuroendocrine carcinoma (SCNEC) is extremely rare, with only one prior case reported, and no site‐specific standard of care has been established. We report a case of primary sublingual gland SCNEC treated with definitive concurrent chemoradiotherapy (CRT). A 69‐year‐old man presented with a 21 × 15‐mm right sublingual mass showing fluorodeoxyglucose uptake (SUVmax 9.9) on PET/CT. No nodal or distant disease was found. Biopsy revealed small cell morphology positive for INSM1/synaptophysin and pan‐cytokeratin, with a high Ki‐67 index (> 95%). IMRT delivered 70 Gy in 35 fractions to the primary tumor with elective bilateral nodal irradiation (levels I–V, 46 Gy). Cisplatin (80 mg/m^2^; day 1) and etoposide (100 mg/m^2^; days 1–3) were administered every 3 weeks for four cycles. Grade 3 oral mucositis and grade 2 neutropenia resolved. At 24 months post‐CRT, the patient remains disease‐free with preserved daily function. Definitive concurrent CRT appears to be a feasible and effective organ‐preserving treatment option for limited‐stage primary sublingual gland SCNEC, with durable disease control at 24 months in this case.

## Key Clinical Message

This case report describes the treatment of a rare primary sublingual gland small cell neuroendocrine carcinoma with definitive chemoradiotherapy. The 69‐year‐old man achieved complete and durable disease control for 24 months, demonstrating the effectiveness of concurrent chemoradiotherapy as an organ‐preserving approach for this exceptionally uncommon malignancy in clinical practice.

## 1 Introduction

Small cell neuroendocrine carcinoma (SCNEC) most often arises in the lung; however, 2%–4% of cases occur at extrapulmonary sites [1, 2, 3]. Among these, head and neck SCNEC is rare and is associated with poor outcomes, with reported 5‐year overall survival rates typically in the 15%–30% range across series, although estimates vary by cohort and site [4]. Because of its rarity, no site‐specific standard of care has been established, and management is generally adapted from small cell lung cancer (SCLC) [4, 5, 6]. Primary SCNEC of the sublingual gland is exceptionally rare; however, to our knowledge, only one case has been previously reported, and durable outcomes after concurrent chemoradiotherapy (CRT) have not been documented. We aimed to describe a case of primary sublingual gland SCNEC successfully treated with definitive CRT.

## 2 Case Presentation

A 69‐year‐old man developed right sublingual pain in April 2023, followed by progressive swelling of the right floor of the mouth by mid‐May. He initially visited a dental clinic and received treatment for apical periodontitis of the right mandibular second molar; however, his symptoms persisted. He was referred to our department in late June 2023 for further evaluation.

**FIGURE 1:**
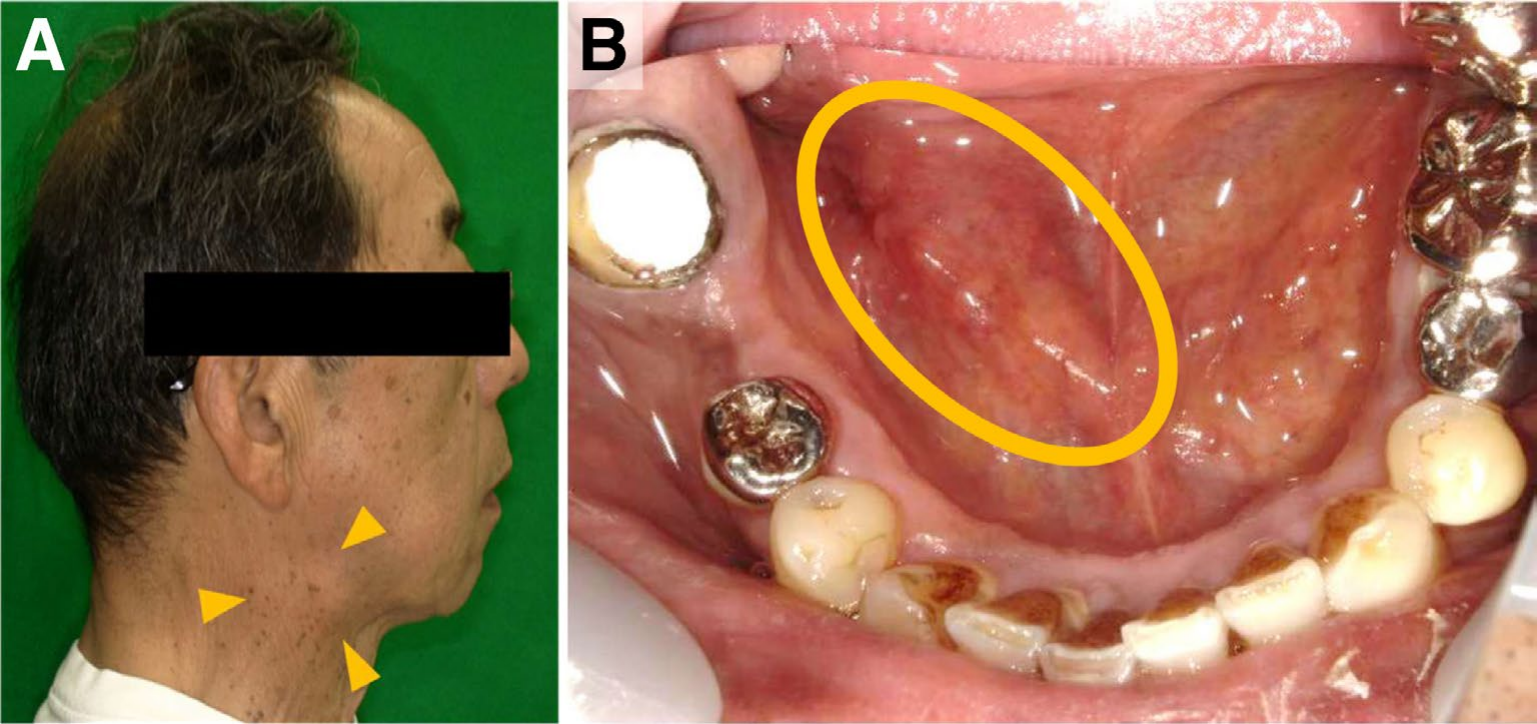
Clinical findings of extraoral and intraoral. (A) Extraoral examination showed tender induration and swelling in the right submandibular area. (B) Intraorally, a poorly circumscribed 30 × 20‐mm submucosal mass was palpable in the right floor of the mouth with normal overlying mucosa; the lesion was elastic‐firm and tender.

At presentation, his general condition was good. Extraoral examination revealed tender induration and swelling in the right submandibular area (Figure 1A). Intraoral examination showed a poorly circumscribed 30 × 20‐mm submucosal mass in the right floor of the mouth with normal overlying mucosa; the lesion was elastic‐firm and tender (Figure 1B). Saliva outflow from the right sublingual caruncle was reduced. His medical history included Hashimoto's thyroiditis and dyslipidemia, with no relevant allergies or family history.

## 3 Imaging Studies

**FIGURE 2:**
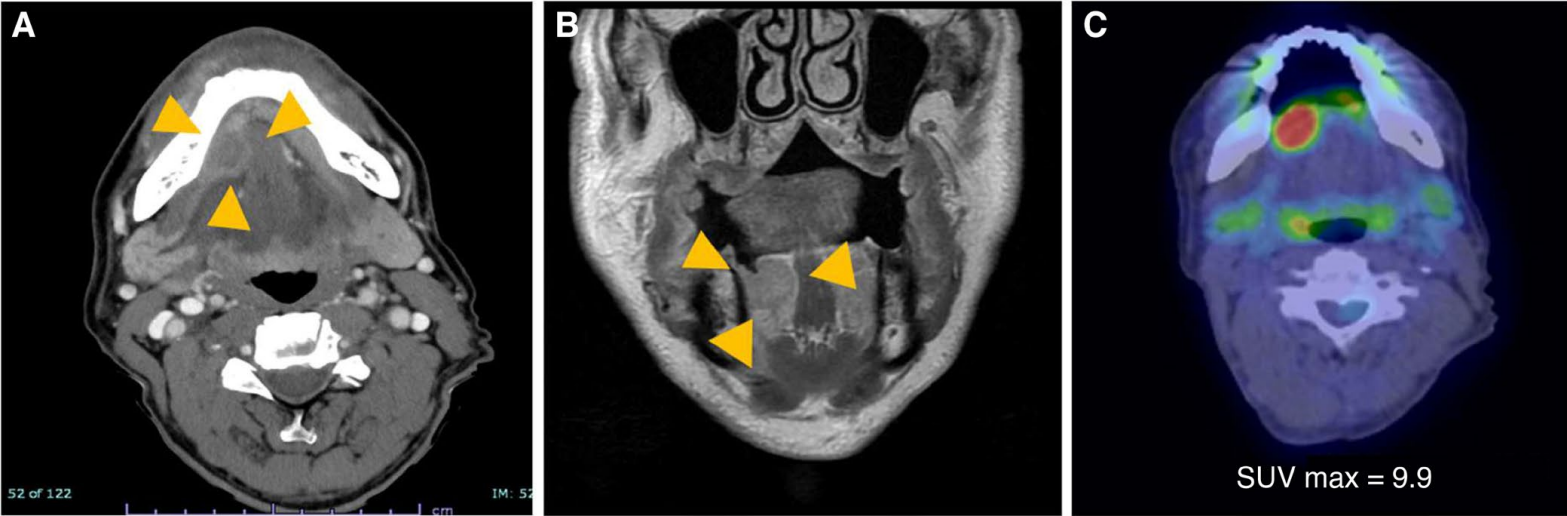
Radiological findings of the tumor. (A) Contrast‐enhanced computed tomography showed a 17 × 16‐mm enhancing mass in the right sublingual region, with no evidence of abnormal cervical lymphadenopathy or pulmonary lesion. (B) Contrast‐enhanced T1‐weighted magnetic resonance imaging showed the right sublingual gland contained a 21 × 15‐mm lesion with abnormal signal. (C) Fluorodeoxyglucose‐positron emission tomography/computed tomography showed intense uptake in the right sublingual region (standard uptake value [SUV] max 9.9) and no abnormal uptake elsewhere.

Ultrasonography revealed a 21 × 15‐mm hypoechoic mass beneath the mucosa of the right floor of the mouth. The mass was irregular in shape, had a slightly indistinct margin, internal heterogeneity, and minimal blood flow signal. Contrast‐enhanced computed tomography (CT) showed a 17 × 16‐mm enhancing mass in the right sublingual region, without abnormal cervical lymphadenopathy or pulmonary lesions (Figure 2A). Magnetic resonance imaging (MRI) demonstrated a 21 × 15‐mm lesion with abnormal signal intensity within the right sublingual gland (Figure 2B). Fluorodeoxyglucose (FDG)‐positron emission tomography/CT (PET/CT) revealed intense uptake in the right sublingual region (maximum standardized uptake value [SUVmax] 9.9) and no abnormal uptake elsewhere (Figure 2C).

## 4 Histopathology and Immunohistochemistry

**FIGURE 3:**
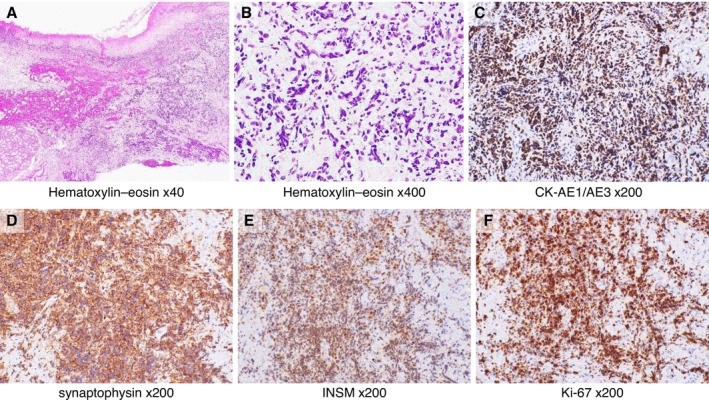
Histopathological findings of the surgical specimen. Hematoxylin–eosin (HE)‐stained section within the salivary gland and overlying oral mucosa, original magnification ×40 (A) and ×400 (B). There was an infiltrative proliferation of atypical cells with high nuclear‐to‐cytoplasmic ratios and hyperchromatic, variably sized irregular nuclei, arranged in solid sheets, trabeculae, and single‐cell patterns; foci of nuclear streaming were noted. (C) Immunohistochemical staining for pan‐cytokeratin (AE1/AE3), original magnification ×200. (D) Immunohistochemical staining for synaptophysin, original magnification ×200. (E) Immunohistochemical staining for INSM1, original magnification ×200. (F) Immunohistochemical staining for Ki‐67, original magnification ×200. Tumor cells exhibited strong cytoplasmic and membranous staining for AE1/AE3 and synaptophysin, with strong nuclear immunoreactivity for INSM1 and Ki‐67.

Hematoxylin–eosin staining showed an infiltrative proliferation of atypical cells within the salivary gland and overlying oral mucosa. The tumor cells exhibited high nuclear‐to‐cytoplasmic ratios and hyperchromatic, variably sized irregular nuclei arranged in solid sheets, trabeculae, and single‐cell patterns, with focal nuclear streaming (Figure 3A,B).

Immunohistochemistry demonstrated positivity for pan‐cytokeratin (AE1/AE3), synaptophysin, and insulinoma‐associated protein 1 (INSM1), and negativity for chromogranin A, cluster of differentiation (CD)3, and CD20. The Ki‐67 labeling index exceeded 95% (Figure 3C–F). Based on the histologic and imaging findings, we diagnosed limited‐stage SNEC of the sublingual gland. Upper gastrointestinal endoscopy performed for staging revealed an erythematous, adenomatous lesion at the esophagogastric junction; biopsy confirmed adenocarcinoma, which was treated after CRT as described below.

## 5 Treatment

A multidisciplinary tumor board recommended definitive concurrent CRT, extrapolating from SCLC management. Intensity‐modulated radiation therapy delivered 70 Gy in 35 fractions to the primary tumor, with elective bilateral nodal irradiation encompassing levels I–V to 46 Gy. Concurrent chemotherapy consisted of cisplatin (80 mg/m^2^, day 1) and etoposide (100 mg/m^2^, days 1–3) every 3 weeks for four cycles. Acute toxicities, graded according to the Common Terminology Criteria for Adverse Events (CTCAE) version 5.0, included grade 3 oral mucositis (necessitating temporary nasogastric feeding) and grade 2 neutropenia after 46 Gy and two chemotherapy cycles, managed with a single dose of granulocyte colony‐stimulating factor (G‐CSF). Despite these toxicities, CRT was completed in late October 2023 without unplanned interruptions, and mucositis resolved within 3 months.

## 6 Outcomes and Follow‐Up

**FIGURE 4:**
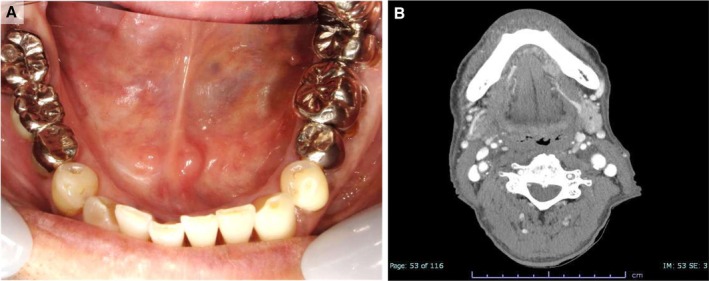
Post‐treatment intraoral and imaging findings. (A) Intraoral examination showed complete resolution of the tumor with no additional mucosal abnormalities. (B) Contrast‐enhanced coronal CT demonstrated no evidence of residual, recurrent, or metastatic disease.

**FIGURE 5:**
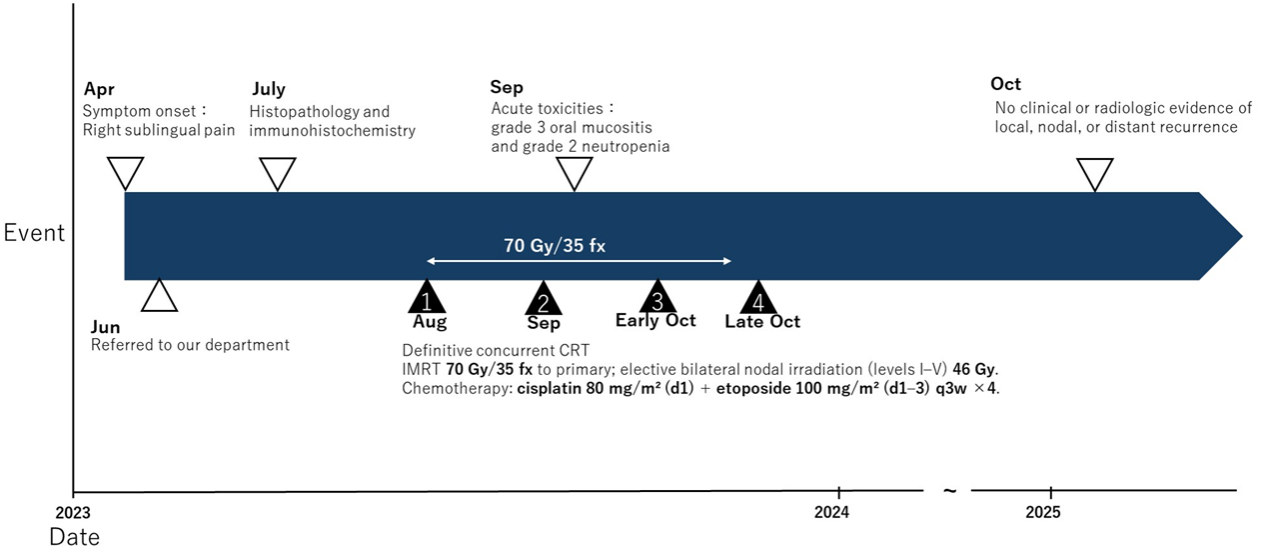
Clinical timeline (CARE‐compliant). Key milestones from symptom onset (April 2023) through definitive CRT (September–October 2023) and 24‐month disease‐free follow‐up (October 2025). Toxicities graded by CTCAE v5.0.

Four months after CRT, with no evidence of recurrence, the patient underwent robot‐assisted proximal gastrectomy with esophagogastrostomy and cholecystectomy for synchronous esophagogastric‐junction adenocarcinoma. Follow‐up evaluations, including clinical examinations, contrast‐enhanced CT, ultrasonography, and laboratory testing, were performed every 2 months. At 24 months post‐CRT, there is no clinical or radiologic evidence of local, nodal, or distant recurrence of SCNEC, and the patient's activities of daily living remain preserved (Figure 4A,B). The patient reported high satisfaction with the treatment outcome (Figure 5).

## 7 Discussion

Extrapulmonary small cell carcinoma (EPSCC) accounts for approximately 2%–4% of all small cell carcinomas, most commonly originating in the genitourinary and gastrointestinal tracts; involvement of the head and neck is rare [1, 2, 3]. Head and neck small cell carcinoma represents only 0.3%–0.4% of all small cell carcinomas [5, 6]. Because of this rarity, no site‐specific treatment standard has been established, and outcomes remain poor, with reported 5‐ and 10‐year overall survival (OS) rates of 27% and 18%, respectively [4]. Moreover, the 5‐year OS for head and neck small cell carcinoma varies by anatomic subsite, generally ranging from approximately 15% to 30% [4].

Management is therefore often adapted from SCLC. Platinum–etoposide is a well‐established regimen for SCLC and can be combined with radiotherapy, with either sequential or concurrent CRT being the standard of care for limited‐stage disease [7]. In limited‐stage SCLC, randomized studies have shown comparable survival and toxicity between radiotherapy of 45 Gy delivered in 30 twice‐daily fractions of 1.5 Gy over 19 days and 66 Gy delivered in 33 once‐daily fractions of 2 Gy over 45 days [8]. The twice‐daily regimen is often selected to shorten overall treatment time. However, when the oral cavity is included in the high‐dose field, accelerated hyperfractionation increases the risk of severe mucositis and unplanned treatment interruptions. Therefore, after multidisciplinary discussion, we selected conventional once‐daily fractions of 2 Gy. Given the biological tendency of SCNEC for early systemic spread and the variable lymphatic drainage from the floor of the mouth, we elected to include bilateral elective nodal irradiation to levels I–V despite negative imaging findings, aiming to minimize regional failure risk.

The role of primary surgery in SCLC remains controversial and may benefit only patients with highly limited stage I–IIA disease. In contrast, several retrospective studies of head and neck small cell carcinoma have shown no OS benefit from adding primary surgery to CRT [4, 9, 10, 11], although these findings are limited by the retrospective design and potential selection bias.

To our knowledge, in the only previously published case of sublingual gland small cell carcinoma [12], surgery was selected for a very old patient (94 years) in whom completing CRT was likely impractical. Postoperative follow‐up was limited to 5 months, and oncologic outcomes were not reported.

Anatomically, the sublingual gland lacks a well‐defined capsule and is prone to extra‐glandular spread. Its proximity to the lingual nerve, submandibular duct, and mandible makes it difficult to achieve wide negative margins while preserving critical neurovascular structures. According to the 5th edition of the World Health Organization Classification Of Head And Neck Tumors, neuroendocrine carcinoma is a poorly differentiated, high‐grade carcinoma diagnosed by morphology and supported by immunohistochemistry findings (such as INSM1, synaptophysin and/or chromogranin A) with typically high proliferative activity (Ki‐67 > 20%, often > 70%) [13]. Our case met these criteria, with a Ki‐67 index of approximately 95%. The primary site was judged to be the sublingual gland because staging imaging did not reveal any pulmonary lesions. Taken together, these considerations supported early initiation of CRT rather than upfront surgery, with comprehensive elective neck irradiation.

Notably, the median OS for oral cavity primary head and neck small cell carcinoma has been reported as 20.8 months [6]. In this context, our patient's durable disease‐free status at 24 months after completion of CRT represents a favorable outcome.

However, head and neck small cell carcinoma carries a high recurrence rate, with distant metastases occurring frequently—particularly when cervical nodal involvement is present. Because control of distant disease is a key determinant of survival in head and neck small cell carcinoma [11], continued vigilant imaging surveillance is essential.

This report has limitations, including its single‐patient nature and the lack of comprehensive molecular profiling beyond immunohistochemistry. Nevertheless, the detailed description of treatment technique, dose, toxicity, and durable disease control contributes valuable information to the sparse literature on sublingual gland SCNEC.

## 8 Conclusion

Definitive CRT achieved complete response and durable disease control at 24 months without functional decline in this patient with primary sublingual gland SCNEC. Given the anatomical challenges of achieving clear surgical margins and the systemic nature of the disease, CRT is a reasonable organ‐preserving option, while surgery may be reserved for highly selected early lesions or for salvage treatment.

## Author Contributions

**Taka‐aki Tokura:** conceptualization, data curation, investigation, visualization, writing – original draft, writing – review and editing. **Akihiro Miyazaki:** methodology, project administration, supervision, visualization, writing – review and editing. **Hironari Dehari:** resources, writing – review and editing. **Koyo Nishiyama:** resources, writing – review and editing. **Sho Miyamoto:** resources, writing – review and editing. **Takanori Sasaki:** resources, writing – review and editing. **Kazuhiro Ogi:** resources, writing – review and editing. **Taro Sugawara:** investigation, validation, writing – review and editing. **Masanori Someya:** resources, supervision, writing – review and editing. **Kohichi Takada:** resources, supervision, writing – review and editing. **Hiroyuki Kitajo:** investigation, resources, writing – review and editing.

## Funding

The authors have nothing to report.

## Ethics Statement

The requirement for ethical approval was waived in accordance with institutional policy; as this single‐patient case report, which describes usual clinical care in an anonymized manner.

## Consent

Written informed consent was obtained from the patient for publication of this case and accompanying images, in accordance with institutional policies.

## Conflicts of Interest

The authors declare no conflicts of interest.

## Data Availability

No new data were generated or analyzed in this study.
